# Sickness-Associated Anorexia: Mother Nature's Idea of Immunonutrition?

**DOI:** 10.1155/2016/8071539

**Published:** 2016-06-29

**Authors:** Gustav van Niekerk, Ashwin W. Isaacs, Theo Nell, Anna-Mart Engelbrecht

**Affiliations:** Department of Physiological Sciences, Private Bag X1, Matieland, Stellenbosch 7600, South Africa

## Abstract

During an infection, expansion of immune cells, assembly of antibodies, and the induction of a febrile response collectively place continual metabolic strain on the host. These considerations also provide a rationale for nutritional support in critically ill patients. Yet, results from clinical and preclinical studies indicate that aggressive nutritional support does not always benefit patients and may occasionally be detrimental. Moreover, both vertebrates and invertebrates exhibit a decrease in appetite during an infection, indicating that such sickness-associated anorexia (SAA) is evolutionarily conserved. It also suggests that SAA performs a vital function during an infection. We review evidence signifying that SAA may present a mechanism by which autophagic flux is upregulated systemically. A decrease in serum amino acids during an infection promotes autophagy not only in immune cells, but also in nonimmune cells. Similarly, bile acids reabsorbed postprandially inhibit hepatic autophagy by binding to farnesoid X receptors, indicating that SAA may be an attempt to conserve autophagy. In addition, augmented autophagic responses may play a critical role in clearing pathogens (xenophagy), in the presentation of epitopes in nonprovisional antigen presenting cells and the removal of damaged proteins and organelles. Collectively, these observations suggest that some patients might benefit from permissive underfeeding.

## 1. Introduction

Infection or tissue trauma is known to induce a range of behavioural modifications collectively referred to as sickness behaviour. Of all these behavioural changes, sickness-associated anorexia (SAA) represents a paradox. Mobilisation of an immune response is metabolically costly [1]. The production of antibodies as well as other signalling peptides (e.g., cytokines and initial phase proteins), expansion of immune cell populations, and the induction of a febrile response all contribute towards a dramatic increase in the demand for metabolic substrates. Yet, despite the cost associated with mobilising an immune response, a decrease in appetite manifests as one of the most cardinal symptoms of an established infection.

Three observations suggest that such SAA represents an adaptive response. Firstly, noninfectious elements such as lipopolysaccharides (LPS) or certain cytokines (e.g., Il-1*β* and TNF-*α*) induce appetite loss [2]. The fact that SAA is engaged by the same canonical inflammatory mediators released in response to infection suggests that SAA forms part of the immune response. Secondly, an array of animals, ranging from vertebrates [3–6] to invertebrates [7–10], all exhibit SAA. Indeed, even primitive animals such as sea anemones retract tentacles and stop feeding after a pathogen challenge [11], indicating that SAA may indeed be evolutionary ancient and conserved across numerous species. In turn, such prevalence amongst animals suggests that SAA must impart a significant fitness advantage. Finally, SAA is consistently observed in a variety of contexts. Only African horse sickness (a lethal vector-borne equine viral infection) is known to induce a fever without inducing an anorexic response [12]. Similarly, a recent review highlighted a number of social factors (e.g., maternal care for the young or guarding behaviour against intruding males) which may attenuate aspects of sickness behaviour [13]. Yet, strikingly, no examples where anorexia is diminished have been identified. Taken together, these observations support the view that SAA is a dedicated host response that facilitates host survival during an infection, though the mechanism by which SAA imparts a survival advantage remains elusive.

A number of theories have been forwarded to explain the adaptive value of SAA. Since free iron is rapidly reduced during an infection [14, 15], it has been argued that chronic low iron levels might provide a pleiotropic benefit by protecting the host against infections [16]. Consequently, an anorexic response may deny pathogens critical resources such as iron [15]. However, recycling of endogenous reserves (e.g., haemoglobin) represents the primary mechanism by which iron levels are maintained, with dietary reabsorption being highly ineffective. In fact, less than 1% of dietary iron is absorbed, with iron status responding only slowly to a changing nutritional status [17]. Aligning behavioural aspects with immunological prerogatives has also been proposed as a physiological strategy pursued by sickness-associated anorexia. In conjunction with a general sense of fatigue, anorexia may decrease an animal's motivation for engaging in foraging behaviour [12]. Also, foraging animals expose a larger surface from which they may radiate heat, thus limiting the efficacy of a febrile response [12]. In addition, infected animals may be less attentive to their surroundings [12] or more conspicuous [18] and accordingly under greater risk of predation. Thus, decreasing foraging activities would possibly reduce exposure to predators. Furthermore, since many pathogens gain entry via the oral route, anorexia may prevent exposure to a fatal pathogen load [12]. Yet, although LPS administration dramatically decreases food intake in rats, hoarding behaviour seems only modestly reduced if under restricted food access (30 min/day) [19]. In fact, Siberian hamsters (which are dedicated hoarders, unlike house mice and rats) injected with LPS demonstrate a dramatic decline in feeding, while hoarding behaviour remains mostly intact [20]. Consequently, it remains to be explained why an animal would demonstrate hypophagia as an energy-conserving or predation-avoiding strategy but remains actively foraging.

We recently argued [21] that a reduced appetite during an infection may represent an evolutionary conserved strategy for systemically upregulating another evolutionary conserved process, autophagy. A brief overview of the ambiguous results on studies investigating the benefit of nutritional support in critically ill patients is provided, followed by an overview of the nutritional context of immune cells during an infection. Next, a summary of the various mechanisms by which an increase in autophagic activity may influence clinical outcome is provided. This is followed by a discussion addressing some of the key limitations of SAA as an inducer of autophagy, with reference to the clinical implication for nutritional support. Finally, we address outstanding questions regarding the role of autophagy, and how insight into these considerations may lead to more refined nutrition support protocols.

## 2. Is Nutritional Support Beneficial?

A major goal of nutritional support in critically ill patients is to avoid the loss of muscle mass, a clinical marker for mortality and morbidity. Yet, various lines of evidence suggest that nutritional support may not provide any benefit, and indeed, may potentially be harmful in certain contexts. In a rat model of septic shock, “immunonutrition” with polyunsaturated fatty acids or arginine aggravated disease progression [22]. Similarly, nutritional intervention has not consistently been demonstrated to be effective in critically ill patients. Indeed, it was found in one study that patients receiving early parenteral nutrition (on day 3 in the intensive care unit) had a small but significantly higher incidence of infection [23] while another study showed a decrease in mortality associated with permissive underfeeding (60–70% of caloric goal) [24]. A recent Cochrane meta-analysis reported that the only benefit associated with nutritional therapy is a decrease in nonelective readmission [25]. Indeed, a consortium of experts have advocated that permissive underfeeding rather than full caloric feeding should be applied to critically ill patients suffering sepsis or septic shock [26]. Yet, the mechanism by which a decrease in nutritional intake would be beneficial remains to be fully elucidated.

A similarly puzzling phenomenon is the paradoxical benefit observed during short-term fasting: starvation, up to three days prior to* Listeria* challenge, reduced the mortality rate to only 5% (compared to a fed group with a mortality rate of 95%) [27]. In addition, starvation promoted macrophage activity against bacteria such as* Listeria monocytogenes* (both in vivo and in vitro), which could be further enhanced by LPS administration [28]. M. J. Murray and A. B. Murray [29] also recount an interesting anecdote provided by Edward [30] who noticed that starved hedge-hogs seemed immune to foot and mouth disease. Correspondingly, force-feeding during an infection resulted in a* higher* mortality rate among mice [29]. Thus, there is both clinical and preclinical evidence indicating that nutritional support does not benefit all patients.

## 3. Starvation: A Calculated Response

It is widely accepted that starvation potently inhibits immune function [31], suggesting that SAA may impede the mobilisation of an effective immune response. Yet, animals have evolved a range of adaptations to cope with nutrition stress [32]. Immune cells in particular occupy a privileged position with regard to the provision of energy-dense substrates. Indeed, during an infection, the expansion of immune effectors is fuelled by peripheral catabolism. In this regard, a number of physiological adaptations ensure that, despite a decrease in feeding, the immune system does not become nutrient deprived.

### 3.1. Energy-Rich Metabolites and Paracrine Signalling

Activated immune cells are highly dependent on glucose. Indeed, hypoxia-inducible factor (HIF), a major inducer of glycolysis, is necessary for macrophage maturation [33]. Conversely, a switch towards oxidative metabolism is accompanied by an activation of an anti-inflammatory programme [34]. It must be noted that although glycolysis is usually active during hypoxia, activated immune cells, similar to other rapidly dividing cells such as cancer cells and proliferating fibroblasts, engage in a form of oxidative glycolysis: these cells produce ATP via glycolysis irrespective of oxygen tension. Such aerobic glycolysis (Warburg effect), which is less efficient than oxidative phosphorylation, is likely explained by two possible factors [35]. First, the inefficiency of glycolysis is compensated for by the rapid speed by which a cell can generate ATP via glycolysis. Second, metabolic intermediates of glycolysis are easily fluxed into biosynthetic pathways that are also upregulated in rapidly dividing cells. As an example, the acetyl-CoA which is required for the synthesis of fatty acids is derived from glycolytic pathway. In this regard, the synthesis of fatty acids is critical for immune cell function. In fact, compromising the ability of monocytes to synthesise fatty acids prevents differentiation into mature macrophages [36]. The dependency of fatty acid synthesis is in turn explained by the demand for phospholipid synthesis: an expansion of cellular components such as endoplasmic reticulum (ER), mitochondrial network, lysosomes, and the development of filopodia all make it necessary for lipids to be incorporated into membrane structures [36]. Thus, activated immune cells require glucose for energy production as well as for the biosynthesis of cellular components. It is thus likely that the Warburg effect can be explained by the fact that glycolysis intersects both energy production and biosynthesis.

A number of key regulatory factors ensure that immune cells are also well supplied with glucose. Proinflammatory cytokines TNF [37] and IL-1*β* [38] induce insulin resistance and thus elevate blood glucose levels. In turn, high glucose levels facilitate the influx of glucose into immune cells via GLUT-1 transporters. Since a GLUT-1 transporter is a facilitative transporter, intracellular glucose is dependent on the extracellular glucose concentration. Thus, elevated glucose levels during an infection are likely to be an adaptive strategy to ensure adequate glucose content in activated immune cells. Similarly, a severe infection is often associated with hypertriglyceridemia. In rats, even low doses of LPS rapidly induce hypertriglyceridemia [39]. High triglyceride levels are maintained through de novo synthesis in the liver [39] and release from adipocytes [40]. Similarly, the development of insulin resistance by LPS binding to TLR4 [41] leads to the secretion of inflammatory cytokines that induce a state of insulin resistance [42]. Thus, infection results in the liberation of energy-rich molecules in circulation which drive immune cell metabolism.

Adipose tissue also seems to be functionally integrated with the immune system, particularly adipocytes which are anatomically associated with lymphoid tissue [43–46]. Since mobilisation of an immune response is costly, special mechanisms exist to suppress immune function during nutrient stress, for example, leptin acting as an “immune-trophic” factor by signalling energy status and thus allowing the optimisation of an immune investment within context of metabolic reserves [47]. In fact, even short-term fasting is able to decrease leptin levels [48]. In this regard, the finely branched lymph vessels increase surface area [44, 45], which facilitates local supply of energy-rich molecules as well as adipocyte-derived paracrine factors [43–45, 49]. Such local paracrine signalling may play an important role during fasting. The finely branched lymph vessels permeating through local adipose deposits represent an anatomical adaptation that may also facilitate paracrine signalling factors between adipocytes and immune cells [43–45, 49]. Supporting this view, adipocytes associated with lymphoid tissue do not respond to normal fasting cues but instead are more sensitive to immune signals, suggesting that these cells are dedicated to assisting immune cells [43–46, 49]. Similarly, bone marrow fat (BMF), whose function remains largely unknown, may function as a “paracrine factory,” sustaining local immune cells even in the face of low nutrient availability. Indeed, BMF is highly unresponsive to fasting and exhibits a paradoxical* increase* in anorexic patients [50]. It is tempting to speculate that an increase of BMF during anorexia (and aged individuals [51]) may be an attempt to counteract immune-antagonising signalling context. The fact that BMF cells are, unlike normal adipocytes, not conglomerated but in fact interspersed, indicating a potential paracrine rather than storage function, supports this view [52]. Finally, ovarian cancer cells metastasising to the omentum (a “sail” of adipose tissue, permeated with lymph vessels) were found to be “fuelled” by adipocytes that directly transfer lipids to cancerous cells [53]. Though these authors identified beta-oxidation as a likely endpoint of the fatty acids, it is also likely that the fatty acids derived from these cells may be involved in the synthesis of cellular components. It is likely that these lymphoid-associated adipocytes may respond to signalling queues usually presented by activated immune cells, which are coopted by cancer cells.

These aforementioned observations suggest that adipocytes may play a critical immune-supporting role by providing energy-rich substrate for both biosynthesis and energy production, as well as paracrine factors for sustaining an immune response within a fasted state. Furthermore, recent findings indicate that the close cooperation between the immune system and adipocytes might extend beyond the simple task of supplying energy-rich molecules and paracrine factors. As an example, adipocytes are known to generate antimicrobial peptides [54] and exhibit a phagocytic capacity [55] and since adipocytes can express MHC II [56], they possibly also play a role in epitope expression. It is thus evident that adipocytes are functionally integrated with the immune system. Indeed, it has previously been argued that the advent of adipocytes, a tissue unique to vertebrates [57], may have allowed the evolution of an adaptive immune system [58]. These adaptations ensure that fasting during an infection would, in an otherwise healthy and well-nourished individual, not impede immune function.

### 3.2. An Altered Amino Acid Profile Leads to an Upregulation of Autophagy

In contrast to glucose and lipids, it has long been recognised that the plasma levels of most amino acids (AAs) undergo a marked decline during a range of different infections or sterile tissue damage [59–64]. Of note, AA levels are in a dynamic state, often exhibiting a rebound effect and are most likely influenced by the severity of an infection or simulation thereof, as well as the time point at which serum AA levels are sampled. Such a decrease in AA occurs in the context of rapid muscle catabolism, suggesting the prime recipients of liberated amino acids may be the liver, where amino acids are used in gluconeogenesis, and immune cells for rapid cell division or anabolism. The observation that serum levels of branched chain AA (BCAA) often decrease during an infection [60, 65–67], in conjunction with ex vivo studies demonstrating the ability of leucine to inhibit muscle degradation [68], initially generated much excitement as a potential supplement in septic patients. Since critically ill patients exhibit an increase in protein turnover [69], these observations suggest that AA supplementation would be beneficial to critically ill patients. However, results from studies applying supplementation with these or other amino acids have been disappointing and there is currently no definitive study that demonstrates the optimum protein provision in critically ill patients [70].

Low AA profiles during an infection thus raise two questions. Firstly, it remains to be explained why AA supplementation, despite the decline in plasma levels of various AA levels at some point during an infection, failed to consistently demonstrate clinical benefit. Secondly, the reason why various AAs decline at some point during an infection is peculiar, given the fact that a range of other sophisticated adaptations exist to supply immune cells with energy-rich substrates for energy production and biosynthesis.

## 4. SAA as an Inducer of Autophagy

### 4.1. SAA Promotes Upregulation of Autophagy

All eukaryotic cells, when starved of nutrients, activate an ancient catabolic process known as autophagy. Autophagy (derived from the Greek words* auto* meaning “self” and* phagy* meaning “eat”) is an evolutionary conserved process by which eukaryotic cells degrade large cellular components into substrates that subsequently can be either used as fuel source, or utilised for the synthesis of critical cellular components. Whereas the proteasome degrade proteins, autophagy is used in bulk degradation of cytoplasmic components, including organelles such as mitochondria or long-lived proteins (process formally known as macroautophagy). Autophagy is rapidly upregulated in starved cells derived from a range of eukaryotes, including plants [71], yeasts [72], invertebrates [73], and mammals [74]. The autophagic process is critical for cell survival during nutrient stress. Transgenic mice with defective autophagic process develop normally but die shortly after birth [75]. Remarkably, it was established that defects in autophagy resulted in these mice being unable to maintain nutrient homeostasis during the transition from placental nutrition to feed-fasting cycles associated with suckling, thus clearly demonstrating the pivotal role of autophagy supplying nutrients during fasting. The evolutionary conserved process of autophagy thus plays a critical role in regulating cellular nutrient status in a fasted state, where it is consistently and robustly upregulated during periods of fasting.

The mechanism by which nutrient stress can induce autophagy has recently been reviewed [76, 77]. Briefly, autophagy is inhibited by mTOR and upregulated by AMPK. A low ATP : ADP ratio results in the activation of AMPK. In turn, activated AMPK inhibits mTOR, a major inhibitor of autophagy. Low energy status is thus one mechanism by which autophagy is upregulated. In this regard, cytokine-mediated insulin resistance may represent a strategy to induce low levels of energy stress in cells, prompting upregulation of autophagy; alternatively, an increase in the cellular AA pool results in the activation of mTOR and a subsequent inhibition of autophagy. As an example, supplementation with BCAA such as leucine protects against muscle wasting induced by a protein-deficient diet [78]. Of note, these authors implicate a decrease in protein degradation, rather than synthesis as the mechanism by which leucine protects muscle mass during AA starvation. This study again emphasises the ability of nutrients such as AA to potently inhibit autophagy. Additionally, depletion of AAs results in the accumulation of unchanged tRNA which in turn also induces autophagy [79]. Finally, under conditions of nutrient deficiency, cells utilise amino acids as a source of energy and metabolic waste products, such as ammonia, accumulate. In this regard, ammonia has also been shown to induce autophagy, but by an mTOR-independent mechanism [80].

**Figure 1:**
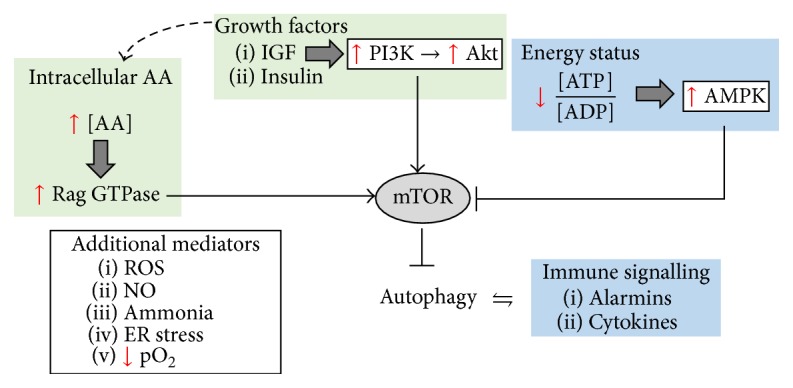
Autophagy plays a key role in energy homeostasis, immune regulation, and a generic stress response to various insults. Growth factor signalling is known to inhibit autophagy directly but may also influence autophagy indirectly, by controlling the cellular import system for AAs. High levels of AAs (in particular, essential AAs such as the BCAA, leucine) also inhibit autophagy through activation of mTOR. In contrast, low energy status upregulates AMPK, which in turn inhibits mTOR, resulting in an increase in autophagy. Autophagy can also be activated by immune effectors including TLR-4 activation or alarmins or via cytokine such as Il1-b and IFN-*γ*. Also, autophagy modulates these inflammatory mediators, for example, by degrading the inflammasome. Finally, autophagy as a generic stress response is also upregulated under hypoxic conditions, or in response to oxidative stress.

Starvation is not the only trigger for autophagy (Figure 1). As an example, ER stress also induces the activation of autophagy [81], where autophagy provides a mechanism for degrading misfolded protein aggregates that cannot be accommodated by the proteasome. In fact, a range of cellular insults including free radicals [82], heavy metals [83], or cytotoxic chemotherapeutics [84] induce autophagy as a general stress response. Also, autophagy is regulated by growth factors [85]. Singling pathways activated by growth factors can directly inhibit autophagy (e.g., Ras activation [86]). Conversely, growth factor withdrawal causes a decreased expression of major nutrient transporters on cell surface, resulting in a decrease in intracellular nutrients, thus leading to the activation of autophagy by nutrient stress. Finally, autophagy is a selective process, being able to target substrates for degradation. As an example, autophagy has been shown to selectively target damaged mitochondria (i.e., mitochondria with a low membrane potential) for degradation, thus playing an important role in mitochondrial “quality control” [87] (a process referred to as mitophagy). The fact that the catabolic process of autophagy can selectively target substrates for degradation also positions autophagy for another key cellular process: host defence against intracellular pathogens. In fact, autophagy is also involved in the degradation of pathogens (formally known as xenophagy) [88].

Therefore, a plausible relationship exists between SAA as a mechanism for upregulating autophagy and the antimicrobial function of autophagy. In fact, the altered AA profile seen in patients may represent a mechanism by which AA withdrawal inhibits mTOR, with a subsequent increase in autophagy. Interestingly, depriving macrophages of growth factors and AAs for four hours upregulated autophagy and provided added protection against* Mycobacterium tuberculosis* (TB) [89]. This suggest that upregulating macroautophagy through fasting can augment immune function.

### 4.2. The Role of Autophagy during an Infection and Tissue Trauma

**Figure 2:**
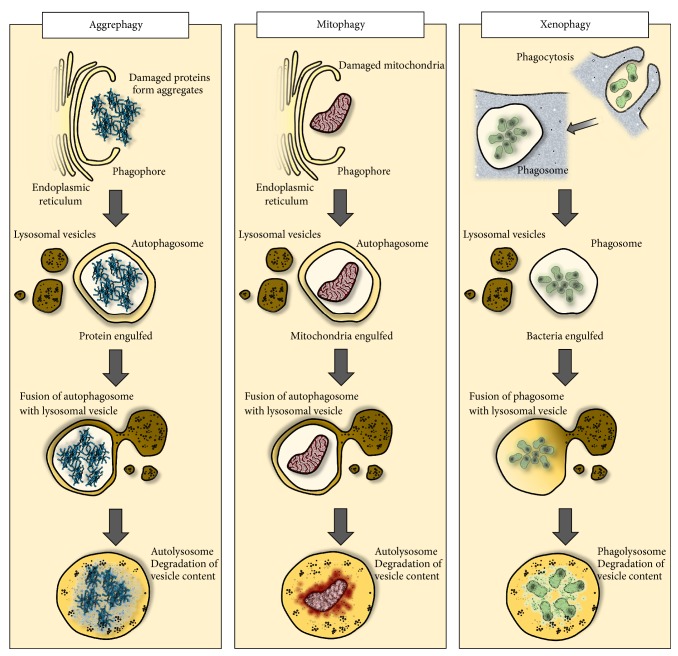
Autophagy plays a critical role in pathogen clearance and host survival. The fibril response as well as oxidative stress resulting from tissue ischemia or immune activation may damage proteins which in turn form toxic aggregates which are cleared by autophagy (“aggrephagy”). Similarly, damaged and dysfunctional mitochondria are targeted for cellular digestion (mitophagy), thus optimising energy generation while diminishing ROS production. Autophagy is also involved in the clearance of intracellular pathogens (xenophagy) in both immune and nonimmune cells. Not demonstrated, autophagic processes are also involved in epitope expression and may provide an alternative form of cell death in viral-infected cells. Also, autophagy may modulate the inflammatory tone by possessing membrane receptors and singling platforms such as the inflammasome.

In previous work, we argued that autophagy has a number of critical functions during an infection [21] (Figure 2). As an example, a higher rate of autophagic flux would confront intracellular pathogens with a shorter time frame for manipulating the host's intracellular defences. Similarly, pathogens have evolved mechanisms for inhibiting apoptosis in response to viral subversion of genomic replication machinery [90]. In this regard, autophagy-mediated cell death (autosis) [91] may provide a “backup” cell death programme. In addition, a range of prominent autophagic mediators (e.g., BECN1, ATG5, ATG7, and ATG12) are also involved in apoptotic pathways [92], suggesting that upregulation of autophagy may “prime” cells for cell death. This would also suggest that SAA may be a protocol not only for enhancing the antipathogen activity of immune cells, but also for cell-autonomous defences, thus obstructing the spread of infectious agents. Finally, autophagy is involved in the expression of epitopes in nonprofessional antigen-presenting cells such as muscle [93], endothelium [94], and adipocytes [54], suggesting that SAA-induced autophagy may augment immune operations by recruiting nonimmune cells in mobilising an adaptive immune response. Supporting this view, inhibition of mTOR, a potent initiator of autophagy, enhances the efficacy of influenza vaccine in mice [95]. Similarly, viral inhibition of the proteasome pathway, a strategy to prevent cells from processing and expressing viral epitopes on MHC I, can be compensated for by autophagic processing of endogenous proteins [96].

Moreover, autophagy may also enhance host survival via the nonimmune mechanism. A severe inflammatory episode challenges cells with a number of insults that must be dealt with. Indeed, the development of clinical hypothermia in severe cases of sepsis may be a strategy to avoid overwhelming of cellular survival systems. Supporting this view, in a rat model of severe endotoxemia, rats that were allowed to develop hypothermia (an observation often seen in cases of severe systemic inflammation) demonstrated a significant increase in survivability [97]. Autophagy as a generic cell survival response may play a critical role in addressing the stressors imposed by a systemic inflammatory context. Indeed, insufficient autophagy in rabbits suffering severe burns has been implicated in mitochondrial dysfunction and organ failure [98]. In addition, autophagy may play a role in clearing phagocytosed apoptotic bodies [99], thus avoiding secondary necrosis and subsequent inflammation. Furthermore, autophagy is also implicated in the degradation of the inflammasome complex [100], thus controlling inflammatory tone and rendering cells more responsive to the disease trajectory. Finally, an increase in body temperature within a fever range induces the expression of heat shock proteins [101], suggesting that newly synthesised proteins may occasionally be misfolded. In this regard, autophagy may play an important role in degrading aggregates of misfolded proteins [102]. In summary, it is evident that autophagy is involved in both pathogen clearance and host survival during an infection.

## 5. Fasting-Induced Autophagy: A Double-Edged Sword?

Host-pathogen coevolution results in the constant development of novel immunological strategies by host, which in turn place evolutionary pressure on pathogens to develop effective counter-measures [103]. In this regard, the fact that autophagy is also upregulated by immunological signals such as LPS and TNF [104] not only emphasises the role of autophagy within an immunological context, but also suggests that pathogens coevolved with the autophagic process as a host defence mechanism. Indeed, pathogens have evolved a number of strategies to subvert the autophagy process [105]. This suggests that fasting may be a strategy to upregulate autophagy as a result of an escalating “arms race” between host and pathogens: by invoking fasting conditions, autophagy is synergistically activated by two independent pathways (immunological activation as well as fasting-induced autophagy). Such a dual activation (by fasting cues and immunological signalling cascades) may render the autophagic process more resistant to “singling attacks” by pathogens attempting to subvert or inhibit autophagy.

However, some pathogens have not only evolved successful measures for subverting host autophagic machinery. This is well exemplified by* Trypanosoma cruzi*. The pathogen's internalisation into mouse embryonic fibroblasts is hampered by deletion of key autophagic proteins, Atg5 and Beclin 1, and similarly is promoted by conditions that enhance autophagy (inhibition of mTOR by both rapamycin and AA starvation) [106]. This indicates that upregulation of autophagy may promote the growth of* Trypanosoma cruzi*. In fact, these authors point out that the disease course of* T. cruzi* infections may be adversely affected by the nutritional state of infected individuals: in undernourished individuals, with high rates of hepatic autophagy, the growth of* Trypanosoma cruzi* may be enhanced. Therefore, it is likely that similar clinical contexts exist in which nutritional support may be implemented in mediating therapeutic inhibition of autophagic processes.

Furthermore, aggressive nutritional support may be necessary in patients who are already undernourished. As mentioned earlier, leptin plays a key role in maintaining immune function. In fact, leptin expression is also markedly increased in response to an immune challenge [107]. Conversely, mice lacking leptin or leptin receptors exhibit defects in their immune function [108]. In this regard, leptin levels parallel body fat mass, indicative of long-term nutritional status [109]. In undernourished individuals, chronically low leptin levels have been implicated in the immune insufficiency observed in these individuals. Thus, a physiological difference between fasting and starvation is a key point that needs to be investigated. Nutritional state prior to an insult may play a critical role in targeting patients that would benefit most from nutritional support, and those who would tolerate permissive underfeeding with a more favourable outcome.

This also points to another key aspect of nutritional support, namely, the timing of nutritional intervention. As argued here, maintaining high levels of autophagy may be advantageous during the initial phases of an infection or injury. However, during the resolution phase, nutritional support may become crucial in sustaining anabolic repair processes. Supporting this view, a recent study in critically ill children [110] has demonstrated superior outcome with late (postponed by 1 week) parenteral nutrition, reaffirming an observation also made for adults [23, 111]. A contributing factor is the possibility that withholding parenteral nutrition during the early phase of an injury/infection may promote higher levels of autophagy when it is most beneficial. In contrast, nutritional supplementation may be more advantageous during the resolution phase, when macronutrients are required for effective tissue repair.

The ambiguous results from clinical trials suggest that some patients may benefit from nutritional support whereas others may in fact be negatively affected by nutritional interventions. Clinical trials are often performed on “critically ill” patients without distinguishing between the nature and the cause of the illness. Reevaluating clinical benefit in the context of different disease settings may point out the context in which preservation of autophagic processes may be more important than supplying metabolic substrates. There is also a need to develop good markers for mapping the disease trajectory, thus allowing for optimised nutritional protocols where nutrients are administered when they are needed most.

## 6. Future Directions

The role of autophagy during an infection, and possibly also in the context of tissue trauma, highlights a number of issues regarding the role of nutritional therapy in critically ill patients. Nutritional support increases circulating AA levels which inhibit autophagy. Similarly, the secretion of bile salts in response to a meal also inhibits hepatic autophagy. This raises the question whether dietary formulation could be designed to supply patients with nutrients without inhibiting autophagic processes. As mentioned previously, bile acids that are reabsorbed after a meal inhibit hepatic autophagy. This would suggest that enteral nutritional formulations that avoid extensive bile flow may avoid downregulation of autophagy. Bile acid release is initiated by the gastric peptide hormone cholecystokinin, which is secreted in response to fatty acids in the gastric tract. However, the length of the fatty acids plays a critical role, as it was found that fatty acids shorter than C12 [112] do not stimulate the secretion of cholecystokinin [112]. This would suggest that short chain fatty acids may provide a source of energy without inhibiting autophagy. The role of dietary composition that may supply metabolic substrates with minimum impact on autophagic processes should therefore be investigated in future studies.

In addition, intensive insulin therapy may also play a role in derailing autophagy. A high blood glucose level is an independent marker for increased incidence of infection and clinical outcome in intensive care patients [113], supporting the notion that insulin therapy may lower blood glucose and enhance survival. However, it is not clear whether high glucose levels represent a more aggravated condition, or whether high glucose levels lead to the development of various pathologies. It was found in earlier studies that controlling glucose levels via aggressive insulin therapy (IT) resulted in clinical benefits in intensive care patients [24]. Yet, a number of concerns have been raised regarding the validity of this trial [114]. Indeed, a subsequent trial was terminated ahead of schedule due to increased adverse effects and in particular an increase in complications resulting from hypoglycaemia in patients receiving IT [115]. In this regard, permissive underfeeding may also avoid further exacerbate already elevated glucose levels without the need for coadministration of insulin. In addition, we suspect that these occasional adverse effects may result from insulin's ability to inhibit autophagy via the mTOR signalling pathway [116]. Thus, permissive underfeeding may represent a superior form of nutritional support by both upregulating autophagy and avoiding the further increasing glycaemic index via nutritional support.

There is also a need to elucidate the numerous roles that autophagy plays in a variety of cells over the course of the disease. Previous studies [117, 118] have identified an early upregulation of autophagy in proliferating T cells. Indeed, inhibition of mTOR results in an enhanced T cell mediated immune response and enhanced immune cell memory [119], clearly implicating the role of mTOR in T cell function. However, a recent study [120] where a conditional knock-out system was deployed, demonstrated, contrary to previous findings, that autophagy is not upregulated in CD8 cells during the initial phase of an infection. From day 8, autophagy is robustly upregulated; yet, at this point (day 8), the virus had already been cleared. Thus, the stimulus for SAA is removed at a time when autophagy is upregulated, indicating that autophagy plays a continuous role in the developing disease state and that SAA is not the only mechanism by which cells induce autophagy. Moreover, the function of autophagy in this context seems to be primarily involved in T cell survival by means of a metabolic shift towards fatty acid metabolism. Thus, although autophagy plays a crucial role in viral control (by promoting cell survival of memory T cells), this process must be independent of SAA and thus does not support an interpretation of SAA as the “autophagic trigger.” Furthermore, it has been demonstrated that T cells exhibit pronounced metabolic plasticity [121], which may assist these cells in surviving the range of environments they encounter as migrating cells. Collectively, these observations suggest that though autophagy has an important function in various immunological contexts, these autophagic processes are active, independent of SAA.

Elucidation of autophagic circuits may also be of interest, both in understanding pathogen subversion and to optimise nutritional support. A low ATP : AMP ratio upregulates AMPK, which upregulates autophagy, whereas low AAs inhibit mTOR, a potent inhibitor of autophagy [122]. Thus, autophagy may be upregulated by inhibition of mTOR, as a result of either AA starvation, or mild energy stress. It is thus possible that autophagy may be preferentially induced by different nutritional triggers, though it should be noted that these processes are not mutually exclusive. It is also well appreciated that animals are capable of “self-medicating” by selecting food sources with specific properties in order to rid themselves of various diseases [123]. Indeed, altered food preference has also been reported in invertebrates. Two groups have independently observed that African armyworms prefer and exhibit increased survival when fed a diet with a higher protein-to-glucose ratio during bacterial [7] or viral [8] infections. This suggests that AMPK-activated autophagy may possibly be more important in invertebrates (whereas AA might be the putative autophagic “trigger” in vertebrates). Alternatively, it is possible that invertebrates, during an infection, metabolise AAs as fuelled source, thus increasing ammonia levels with a subsequent increase in autophagy [80]. In fact, a similar mechanism has been observed in a clinical setting: it was shown that LPS impaired the hepatic removal of ammonia via ureagenesis, which might explain the increase in ammonia observed in patients during sepsis [124]. It is tempting to speculate that such a “metabolic dysfunction” is in fact a strategy to elevate ammonia levels slightly, thus increasing the stimuli for upregulating autophagy systemically. Future studies may elucidate the functional value of substrate utilisation, as well as the accumulation of metabolic waste or intermediates in regulating autophagic processes.

Finally, the fact that pathogens have evolved strategies for subverting autophagic process necessitate careful study designs that measure autophagy flux [125]. However, an increase in autophagic elements such as vesicles may indicate an increase in autophagic activity. It may also signify a defect in the autophagic process, such as an inability of lysosomes to fuse with phagosomes, and it may be a direct result of pathogen subversion of autophagic process. Moreover, various proteins involved in autophagy are also degraded by the autophagic process itself [126]; thus, immunoblotting of key proteins may give rise to misleading results.

## 7. Conclusion

The prevalence of SAA across various species suggests that avoiding feeding during an infection is an evolutionary conserved response and consequently must represent an adaptive response. Yet, the metabolic demand of mobilising an immune response motivates the implementation of aggressive nutritional support. Clinical trials and results from animal studies have not consistently demonstrated an advantage of nutritional supplementation. Similarly, as outlined in our introduction, a mechanistic justification for SAA as a beneficial process has not been conclusively identified. The role of SAA as a strategy for upregulating autophagy not only provides a rational basis for explaining the clinical value of permissive underfeeding, but also explains the contrasting results between studies: some patients may benefit from autophagy-mediated cell survival and the antipathogenic effects of this process, whereas some would not. Indeed, in the context of some pathogens, upregulating autophagy may in fact be detrimental. Stratifying patient groups according to the trauma or infectious agents involved may provide insight into the context in which aggressive nutritional support may be warranted and where permissive underfeeding may provide a superior strategy. Finally, there is a need to optimise scheduling when nutritional support is introduced. It is likely that patients would benefit most from nutritional support if administered during the resolution phase, where a shift from catabolic to anabolic metabolism is observed.
